# The dynamics of DNA methylation fidelity during mouse embryonic stem cell self-renewal and differentiation

**DOI:** 10.1101/gr.163147.113

**Published:** 2014-08

**Authors:** Lei Zhao, Ming-an Sun, Zejuan Li, Xue Bai, Miao Yu, Min Wang, Liji Liang, Xiaojian Shao, Stephen Arnovitz, Qianfei Wang, Chuan He, Xuemei Lu, Jianjun Chen, Hehuang Xie

**Affiliations:** 1 Laboratory of Genome Variation and Precision Biomedicine, Beijing Institute of Genomics, Chinese Academy of Sciences, Beijing 100101, China;; 2 Epigenomics and Computational Biology Lab, Virginia Bioinformatics Institute, Virginia Tech, Blacksburg, Virginia 24060, USA;; 3 Section of Hematology/Oncology, Department of Medicine, The University of Chicago, Chicago, Illinois 60637, USA;; 4 Department of Chemistry and Institute for Biophysical Dynamics, The University of Chicago, Chicago, Illinois 60637, USA;; 5 Department of Biological Sciences, Virginia Tech, Blacksburg, Virginia 24060, USA

## Abstract

The faithful transmission of DNA methylation patterns through cell divisions is essential for the daughter cells to retain a proper cell identity. To achieve a comprehensive assessment of methylation fidelity, we implemented a genome-scale hairpin bisulfite sequencing approach to generate methylation data for DNA double strands simultaneously. We show here that methylation fidelity increases globally during differentiation of mouse embryonic stem cells (mESCs), and is particularly high in the promoter regions of actively expressed genes and positively correlated with active histone modification marks and binding of transcription factors. The majority of intermediately (40%–60%) methylated CpG dinucleotides are hemi-methylated and have low methylation fidelity, particularly in the differentiating mESCs. While 5-hmC and 5-mC tend to coexist, there is no significant correlation between 5-hmC levels and methylation fidelity. Our findings may shed new light on our understanding of the origins of methylation variations and the mechanisms underlying DNA methylation transmission.

DNA methylation is a heritable epigenetic mark crucial for diverse biological processes, including transcription regulation and mRNA splicing (Shukla et al. 2011; Jones 2012). The faithful maintenance of methylation patterns is of vital importance and aberrant DNA methylation is frequently observed in tumors and many other diseases (Jones and Baylin 2007). In mammals, the addition of methyl groups onto cytosine residues is directly catalyzed by three DNA methyltransferases: DNMT1, DNMT3A, and DNMT3B (Law and Jacobsen 2010). With the assistance of DNMT3L, DNMT3A/3B can catalyze de novo DNA methylation (Cheng and Blumenthal 2008). During mitosis, DNMT1 is recruited to a DNA replication fork and faithfully copies methylation status from mother strands to daughter strands (Goyal et al. 2006). The suppression of DNMT1 results in unmethylated daughter strands and gradual methylation loss following each cell cycle. The loss of DNA methylation may also occur actively in both dividing and nondividing cells. Although the mechanism of active DNA demethylation remains elusive in mammalian cells, recent studies suggest a potentially active DNA demethylation pathway via the addition of a hydroxyl group onto methylated cytosine and subsequent oxidation mediated by TET enzymes (Tahiliani et al. 2009; Hackett et al. 2013).

Regardless of the high accuracy of DNMT1 and tightly controlled DNA methylation mechanisms, within a cell population, methylation patterns often show molecule-to-molecule variation, and a substantial portion of hemi-methylated CpG dyads have been speculated (Bird 2002; Fu et al. 2010). Such variation in the fidelity of methylation transmission has been associated with the diversity of genomic context and different levels of DNA methylation across the genome (Ushijima et al. 2003). Using an elegant hairpin-bisulfite PCR technique, Laird and colleagues examined two FMR1 alleles in human lymphocytes and estimated 83% as the fidelity of inheritance for the unmethylated cytosine in a hypermethylated CGI but 99% fidelity in the hypomethylated CGI (Laird et al. 2004). Recently, a similar approach was applied to four single-copy genes and several repetitive elements (Arand et al. 2012). The percentage of hemi-methylated CpG dyads varies considerably among these genomic segments and different cell types. On a genome-wide scale, extremely high fidelity on DNA methylation transmission was found for ∼30% of genomic segments derived from CGIs and 10% of segments from *Alu* repeats, whereas only a small subset of CGIs and *Alu* elements have highly variable methylation patterns (Xie et al. 2011). Notably, the methylation fidelities of CGIs and repetitive elements decrease in tumor tissues (Ushijima et al. 2005; Watanabe et al. 2006; Xie et al. 2011). Epigenetic instability in embryonic stem cells has also been well documented (Humpherys et al. 2001; Minoguchi and Iba 2008). Although the cause-and-effect relationships between tumorigenesis and epigenetic instability remain elusive, epigenetic heterogeneity may give stem cells a selective advantage but with an increased risk of tumor formation. Interestingly, during early differentiation, the pluripotent cells gradually develop the capacity to faithfully transmit epigenetic information to their offspring (Skora and Spradling 2010).

Despite decades of effort, current understanding of DNA methylation inheritance is inferred from single strand data or hairpin bisulfite sequencing data for very limited genomic loci. In this study, we aimed to gain a genome-scale view of the dynamics in DNA methylation inheritance and define the factors associated with methylation fidelity. Using mouse embryonic stem cells (ES-E14TG2a) in both undifferentiated and differentiating states as a model system, we implemented a genome-scale hairpin bisulfite sequencing approach to capture the methylation pattern variation during the stem cell transition from self-renewal to commitment, and integrated hairpin bisulfite sequencing data with various “omics” data to scrutinize the relationships among DNA methylation inheritance, gene expression, histone modification, transcription factor (TF) binding, and distribution of 5-hydroxylmethylation cytosine.

## Results

### Strategy for genome-scale hairpin-bisulfite sequencing

**Figure 1.**
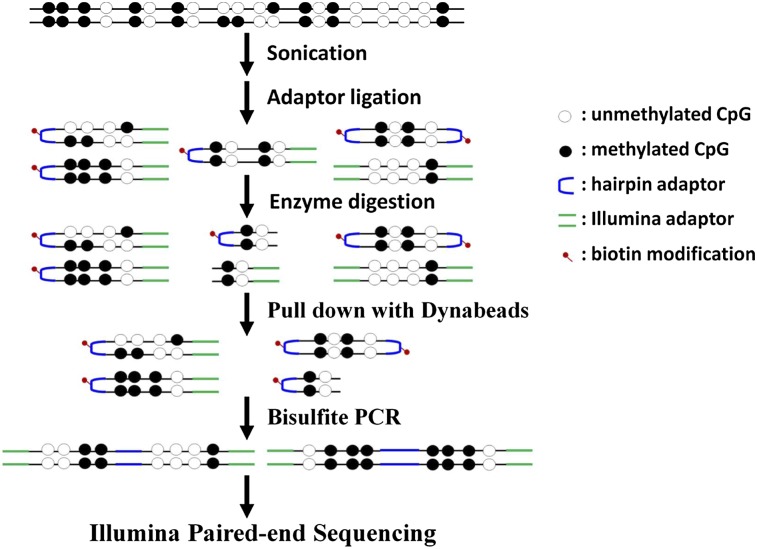
A schematic diagram for genome-scale hairpin bisulfite sequencing.

Epigenetic programming plays an essential role in regulating the balance between stem cell self-renewal and differentiation. Spontaneous differentiation of ES cells can be triggered by the withdrawal of leukemia inhibitory factor (LIF) and dramatic transcriptional changes occur in the very early stages (Williams et al. 1988; Heo et al. 2005; Walker et al. 2007). To investigate the DNA methylation inheritance during ES cell fate commitment, we generated genome-scale hairpin bisulfite sequencing data for the self-renewal and spontaneous differentiating mouse ES-E14TG2a cells (denoted as E14-d0 at day 0 and E14-d6 at day 6 after the withdrawal of LIF, respectively). The differentiation characterization of mES cells was conducted with SSEA-1 (stage-specific embryonic antigen-1) staining, quantitative RT-PCR, and transcriptional profiling of embryonic stem cell markers (Supplemental Fig. S1). Isolated from E14-d0 or E14-d6 cells, genomic DNA was sonicated into fragments ∼200 bp and ligated to the biotinylated hairpin and Illumina sequencing adaptors simultaneously. Restriction endonuclease digestion with MseI (T|TAA) and MluCI (|AATT) was performed to enrich the CG-rich fragments. Following the streptavidin-capture and bisulfite PCR, only the fragments linked to both the hairpin adaptor and Illumina sequencing adaptor were amplified for high-throughput paired-end sequencing (Fig. 1).

### Methylation level and fidelity increase in the early stage of mES cell differentiation

The details of sequencing and mapping results are provided in Supplemental Table S1. A total number of 266.1 and 273.8 million uniquely aligned reads were generated to cover 49.8% and 41.6% of cytosines in CpG context for E14-d0 and E14-d6 cells, respectively (Supplemental Fig. S2). The bisulfite conversion rates were determined to be 98.9% and 98.8% for E14-d0 and E14-d6 data sets, respectively, with the spike-in control lambda DNA. We focused on the analysis of 158,558,697 cytosines with at least 10× sequencing depth (≥5 reads on both strands) for both E14-d0 and E14-d6. Among these cytosines, 7,583,856 are located in CpG context, 35,228,969 in CHG context, and 115,745,872 in CHH context. They represent 17.3%, 16.0%, and 13.8% of the total number of CpG, CHG, and CHH sites in the mouse genome, respectively.

We first analyzed the methylation levels (ML) of CpG and non-CpG sites in E14-d0 and E14-d6. Similar to previous reports (Ramsahoye et al. 2000; Lister et al. 2009; Xie et al. 2012), we detected abundant DNA methylation calls (8.0%) in non-CpG context in E14-d0 and the number decreased to 4.3% in E14-d6 (Supplemental Fig. S3A,B). Most cytosines in CpG context are highly methylated in both E14-d0 and E14-d6, and their methylation levels are characterized by a bimodal distribution (Supplemental Fig. S3C,D). The average methylation level of CpG dyads is 86.3% in E14-d0, and increases to 91.5% in E14-d6 (Supplemental Fig. S3C,D). In contrast, most cytosines in non-CpG context are either unmethylated or lowly methylated (Supplemental Fig. S3E–H).

We further determined the methylation fidelity (MF), as defined in the Methods section, which represents the percentage of symmetrically methylated or unmethylated CpG dyads for a given position. A symmetrical methylation status of CpG dyad (either methylated or unmethylated) indicates successful methylation inheritance, whereas an asymmetrical methylation status (hemi-methylated CpG dyad) indicates gain or loss of the methylation pattern. Most CpG dyads have high methylation fidelity and the average methylation fidelity is as high as 88.5% and 91.9% for E14-d0 and E14-d6, respectively (Supplemental Fig. S4). Although most CpG dyads maintain similar methylation levels and fidelities during mES cell differentiation, our results indicate that both the methylation level and fidelity increase for a considerable fraction of CpG dyads in E14-d6 (Supplemental Fig. S5). More specifically, 24.7% of CpG dyads (17.0% increased, and 7.7% decreased) have methylation level changes >20% (Supplemental Fig. S5C), and 27.5% of CpG dyads (17.3% increased, and 8.3% decreased) have methylation fidelity changes >20% (Supplemental Fig. S5D).

### Methylation level and fidelity vary at distinct genomic regions

**Figure 2.**
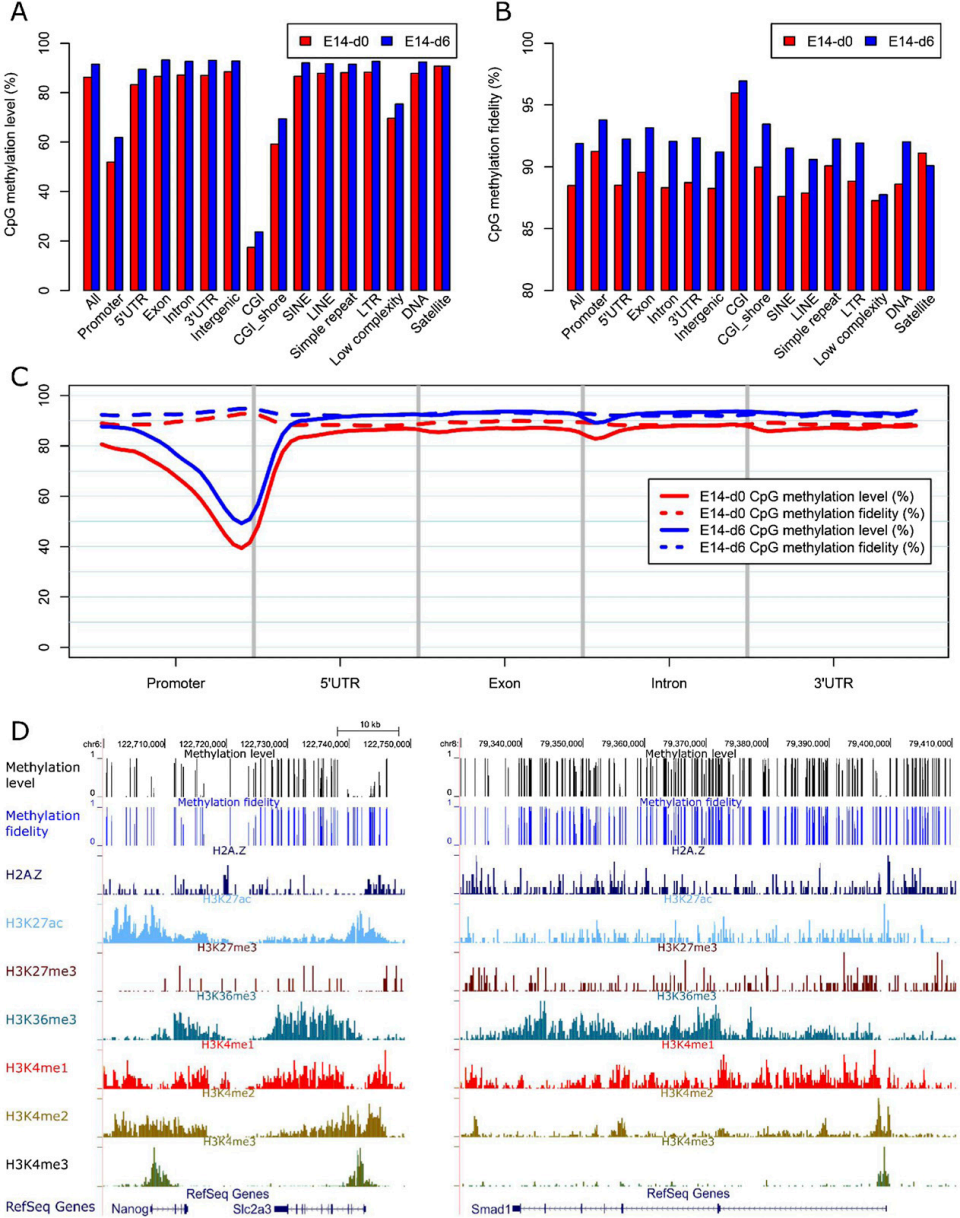
Characteristics of DNA methylation level and fidelity for CpG dyads at different genomic regions. (*A*,*B*) Bar plots showing the methylation level (*A*) and fidelity (*B*) of CpG dyads at different genomic regions. (*C*) CpG methylation level and fidelity along different gene-associated regions. The smoothed lines represent the mean methylation level (solid lines) and fidelity (dashed lines). (*D*) Genome browser representation of methylation level, methylation fidelity, and various histone modifications at genes including *Nanog*, *Slc2a3*, and *Smad1*.

Numerous studies indicate that DNA methylation levels tend to vary across distinct genomic regions (Doi et al. 2009; Lister et al. 2009; Laurent et al. 2010). Consistently, we observed low methylation levels in promoter regions, and the methylation levels are highly correlated with the distance to transcription starting sites (Fig. 2). Low methylation levels were also observed for CGIs and CGI shores (*P* < 2.2 × 10^−16^, Wilcoxon rank sum test), which are frequently associated with promoters in mammals (Supplemental Figs. S6A, S7A). Interestingly, we also observed high methylation fidelity in promoter regions and CGIs (Fig. 2; Supplemental Figs. S6B, S7B). In particular, the methylation fidelity in CGIs is >5% higher compared with the genome-wide average (*P* < 2.2 × 10^−16^, Wilcoxon rank sum test). We further analyzed the CGIs in terms of their CpG densities, and found that the methylation levels of CGIs are negatively correlated with their CpG densities (Supplemental Fig. S7C,D); however, no obvious correlation was observed between their methylation fidelity and CpG density (Supplemental Fig. S7E,F).

In ES cells, significant variations have been observed for the methylation fidelities of different types of repetitive elements (Arand et al. 2012). We found a consistent methylation level across different classes of repeat elements in both E14-d0 and E14-d6, except for “low-complexity,” which showed lower methylation levels (Fig. 2A; Supplemental Fig. S6A). The decreased methylation level for “low-complexity” repeats is probably because of high CpG density (Su et al. 2012) and the overlapping with CGIs. However, the methylation fidelities vary among these repetitive elements. For instance, the average methylation fidelity in “satellite” is 3.9% higher than that of “low-complexity” in E14-d0 (Fig. 2B). In addition, repetitive elements tend to have increased methylation fidelity in E14-d6, but the magnitudes of fidelity changes vary among repeat classes. The methylation fidelity is increased by 3.9% for SINE, while decreased by 1.0% for “satellite” from E14-d0 to E14-d6 (Fig. 2B; Supplemental Fig. S6B). This suggests the dynamics on the activity and genome distribution of DNMTs during early differentiation.

### Bimodal distribution of DNA methylation fidelity

Many genome-wide studies revealed that a significant fraction of CpG dyads exhibit a partial methylation pattern in a given cell population. Such methylation variation may be originated from methylation difference (a) between two strands of the same DNA molecule, (b) between two parental alleles within the same cell, (c) among DNA molecules in different cells, or (d) the combinations of all the above. Apparently, the methylation level and fidelity are closely related. The completely methylated or unmethylated status indicates 100% accuracy on methylation transmission. However, irrespective of methylation levels, methylation fidelity would be the lowest for case (a) and the highest for case (b) or (c).

**Figure 3.**
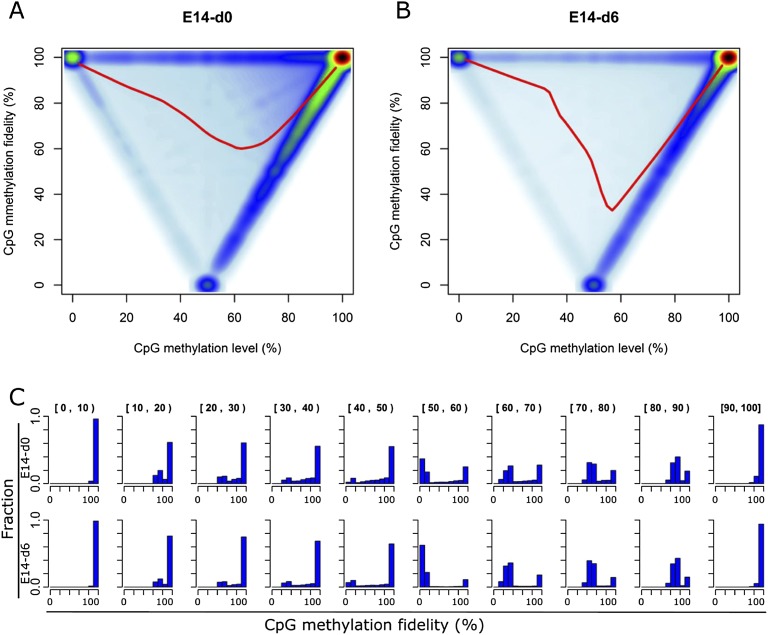
CpG methylation fidelity follows a bimodal distribution. (*A*,*B*) Scatter plots showing the relationship between methylation level and methylation fidelity in E14-d0 (*A*) and E14-d6 (*B*). The smoothed lines represent the mean methylation fidelity along the change of methylation level. (*C*) Histograms showing the distribution of methylation fidelity for CpG sites with methylation levels at 10 intervals in E14-d0 and E14-d6, respectively. The methylation level intervals are indicated in brackets on the *top* of each subplot.

To better illustrate the relationship between the methylation levels and fidelity, we explored their global correlation on a genome-wide scale (Fig. 3). To our surprise, the methylation fidelities of partially methylated CpG dyads follow a bimodal distribution in both E14-d0 and E14-d6 (Fig. 3A–C). For a given CpG dyad, the methylation fidelity tends to be either near the theoretical minimum value (case a) or 100% (case b or c). For instance, ∼15.6% of half-methylated (50% methylation level) cytosines have methylation fidelity at 100%, and 65.2% of these cytosines have methylation fidelity at 0%. In addition, such “bimodal distribution” of methylation fidelity is present among various genomic regions (Supplemental Fig. S8). According to the methylation levels, we further divided the CpG dyads into 10 intervals. As expected, for CpG dyads in the intervals with the lowest (0%–10%) or highest (90%–100%) level of methylation, at least 90% of them tend to have 100% methylation fidelity. For CpG dyads with methylation levels between 10% and 50%, at least 60% of them also have methylation fidelity >90%; however, for those with methylation levels between 50% and 90%, <30% have methylation fidelity >90% and most of the rest have methylation fidelity near the theoretical minimum value (Fig. 3C). Such difference suggests that DNA methylation inheritance of hypo- (i.e., 10%–50% methylated) and hypermethylated (i.e., 50%–90% methylated) CpG dyads may be under the control of different regulatory mechanisms.

We further investigated the spatial correlation of methylation fidelity for four groups of CpG dyads: completely unmethylated, completely methylated, and 50% methylated with 0% or 100% fidelity. Compared with the other groups, genomic regions flanking the unmethylated CpG dyads have the lowest methylation levels and the highest methylation fidelity (Supplemental Fig. S9). Substantially lower methylation fidelity was observed for the genomic regions immediately flanking the hemi-methylated CpG dyads (methylation level = 50% and methylation fidelity = 0%), but such trends fade rapidly over a very short distance. Interestingly, genomic regions flanking these hemi-methylated CpG dyads have significantly higher methylation levels than the ones adjacent to half-methylated CpG dyads but with 100% methylation fidelity. We further determined the nucleotide frequencies in flanking sequences for these CpG dyads (Supplemental Fig. S10). Compared with the other three groups, the completely unmethylated CpG dyads tend to reside in GC-rich regions as expected. For the two groups of half-methylated CpG dyads, no obvious preferences on flanking sequences were found. This is consistent with previous findings that DNMT1 methylates hemi-methylated DNA with no preference on flanking sequence (Vilkaitis et al. 2005; Goyal et al. 2006).

Next we analyzed the CpG density of the flanking 100 bases and the evolutionary conservation of the CpG dyads (Supplemental Fig. S11A–D). In both E14-d0 and E14-d6, the unmethylated CpG dyads tend to localize in regions of high CpG obs/Exp (*P* < 2.2 × 10^−16^, Wilcoxon rank sum test) and the lowest CpG density was observed for the flanking sequences surrounding the half-methylated CpG dyads with 0% methylation fidelity. In addition, unmethylated CpG dyads have a much higher phastCons score (*P* < 2.2 × 10^−16^, Wilcoxon rank sum test) indicating higher levels of conservation. We further examined whether CpG dyads with high methylation fidelity tend to be enriched in certain genomic regions. In both E14-d0 and E14-d6, the CpG dyads with high fidelity are more frequently observed in gene-associated regions including promoters, 5′ UTRs, and exons (Supplemental Fig. S11E,F). Compared with those in E14-d0, the half-methylated CpG dyads with high fidelity in E14-d6 cells show threefold enrichment in promoter regions. This probably results from the increased cellular heterogeneity during cell differentiation.

### Methylation level and fidelity are associated with gene expression, histone modification, and the binding of TFs

**Figure 4.**
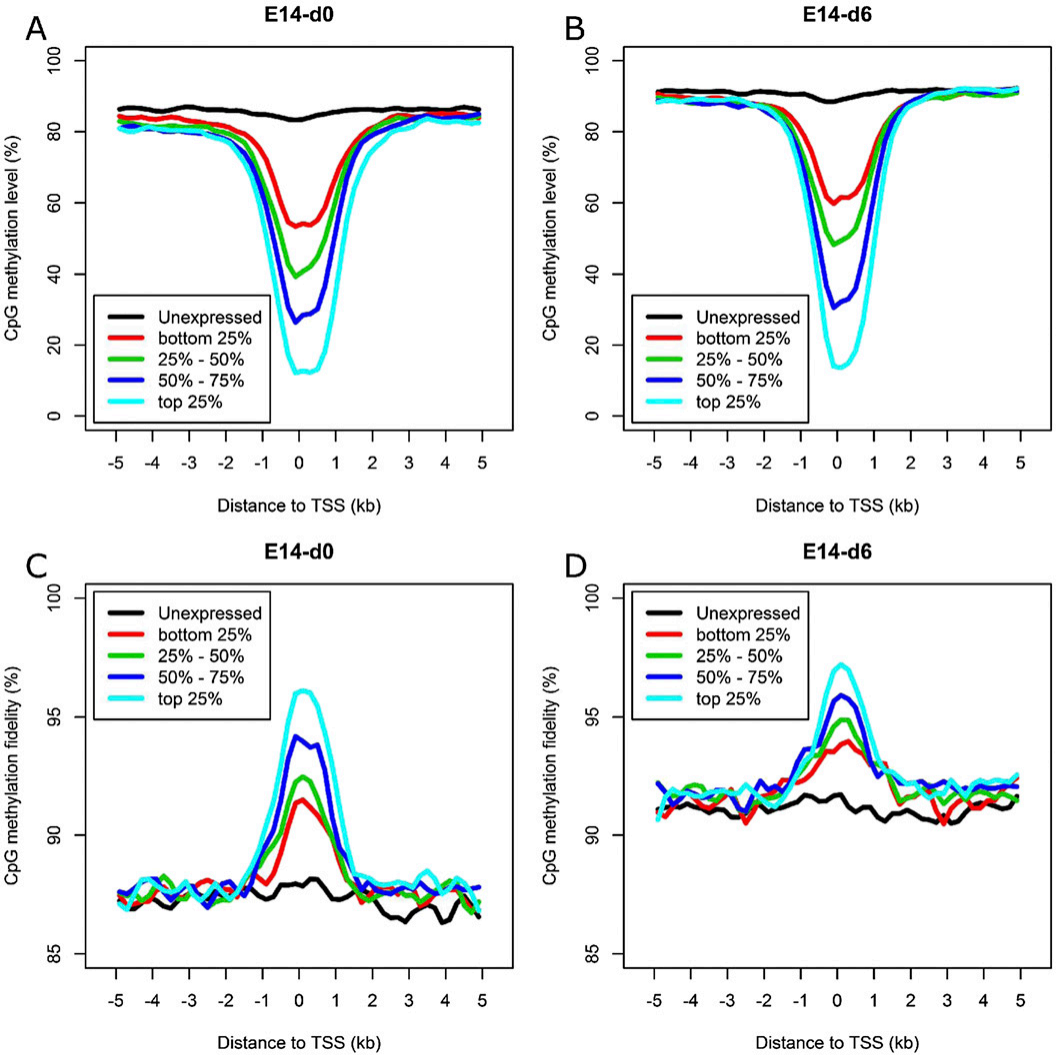
Relationship between DNA methylation and gene expression. (*A*,*B*) Average methylation level of the promoters for genes ranked by expression level in E14-d0 and E14-d6. (*C*,*D*) Average methylation fidelity of the promoters for genes ranked by expression level in E14-d0 and E14-d6. The smoothed lines represent the average methylation level and fidelity surrounding TSSs calculated using 200-bp sliding windows.

Gene expression is tightly regulated by multiple layers of mechanisms, including DNA methylation, histone modification, and transcription factor (TF) binding. To explore the relationship between gene expression and DNA methylation in gene promoters, we classified genes into five groups ranked by expression level. It is obvious that the methylation level of CpGs in promoters is anti-correlated with gene expression (Fig. 4A,B), while methylation fidelity is positively correlated with gene expression (Fig. 4C,D). Interestingly, for genes with no detectable expression, the methylation levels and fidelity surrounding TSSs show nearly no difference compared with distant regions. In contrast, for expressed genes, even of low expression level, their promoters show significantly decreased methylation levels and increased methylation fidelity compared with distant regions. The overall methylation tendencies surrounding TSSs do not differ much between E14-d0 and E14-d6, but remarkable increases in methylation level and fidelity were observed in differentiating cells, in particular for regions distant from TSSs.

**Figure 5.**
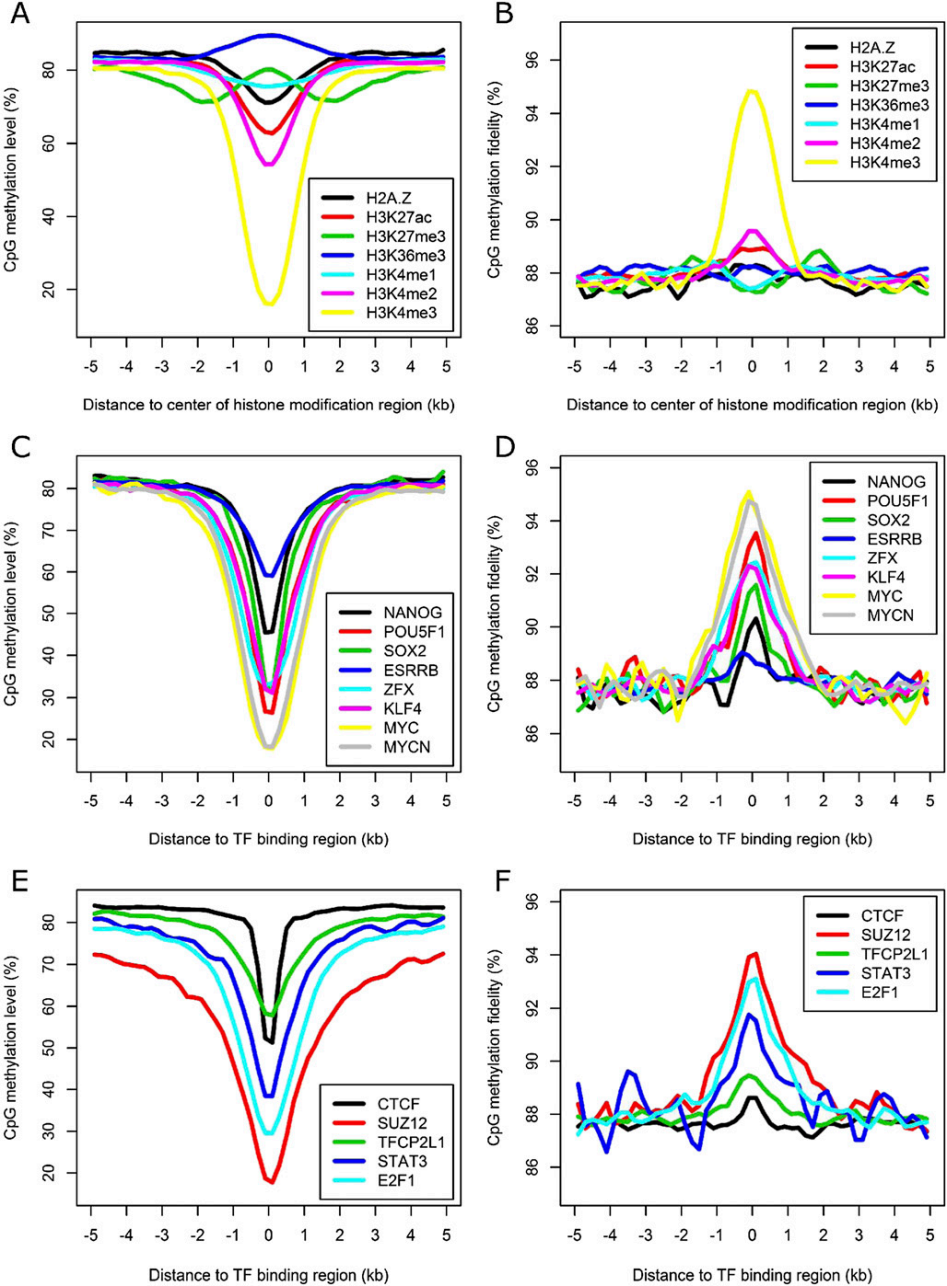
DNA methylation level and fidelity at regions with various histone modifications and TF binding in E14-d0. (*A*,*B*) Profiles of methylation level and fidelity of regions enriched for various histone modifications. (*C–F*) Profiles of methylation level and fidelity surrounding the binding regions of various TFs or regulators. The smoothed lines represent the average methylation level and fidelity surrounding the center of various histone modifications (*A*,*B*) and TF-binding regions (*C–F*), which were calculated using 200-bp sliding windows.

Histone methylations direct the establishment of specific DNA methylation patterns; on the other hand, DNA methylation might serve as a guide for histone modifications after DNA replication (Ooi et al. 2007; Cedar and Bergman 2009). To better understand how DNA methylation inheritance may be affected by histone modifications, we examined the patterns of DNA methylation surrounding genomic regions with various types of histone modifications by integrating methylation data with ChIP-seq data generated from undifferentiated mES cells (Xiao et al. 2012). Consistent with previous reports (Kelly et al. 2010; Lee et al. 2012), we observed low methylation levels in regions enriched with active chromatin mark H3K4me3 and increased methylation levels in regions enriched with the repressive histone mark H3K27me3 (Fig. 5A). Compared with those of the corresponding adjacent regions, the methylation fidelities were moderately increased for regions enriched with H3K4me2 and H3K27ac, and remarkably increased for regions enriched with H3K4me3 (Fig. 5B).

DNA methylation affects the formation of chromatin and the interaction between DNA-binding proteins and their target sequences (Bell and Felsenfeld 2000; Hark et al. 2000). We examined the methylation levels and fidelity surrounding the binding region of 13 TFs, including those important for the pluripotency of ES cells. In general, the binding regions of all factors tend to have low methylation levels and high methylation fidelities, although such correlations fluctuate significantly among these proteins (Fig. 5C–F). We further checked the methylation level and fidelity surrounding the corresponding binding regions of these proteins in E14-d6, and found that the peaks of methylation levels and fidelities of the binding regions of several TFs vital for ES pluripotency, including NANOG, POU5F1, SOX2, and KLF4, are much less prominent than those in E14-d0 (Supplemental Fig. S12). In contrast, considerably fewer changes were observed for the peaks of methylation levels and fidelities of the binding regions of the TFs regulating cell cycle.

We extended the analysis to the four groups of CpG dyads previously selected (Supplemental Fig. S13). The completely unmethylated CpG dyads show ∼18-fold enrichment in regions marked by H3K4me3 but depleted in regions marked by H3K36me3. In addition, the completely unmethylated CpG dyads show six- to 19-fold enrichment in various types of TF-binding sites. This supports our previous observation that these unmethylated CpG dyads have a higher tendency to reside within active promoter regions but not in gene bodies. In contrast, the fully methylated CpG dyads are enriched in regions with H3K36me3 marks and almost completely depleted from TF-binding sites. Interestingly, for the half-methylated CpG dyads, the ones with high fidelity are more likely to be present within genomic regions with active histone modifications, especially H3K27ac and H3K4me1 (Supplemental Fig. S13A). Since H3K27ac marks distinguish active and cell type-specific enhancers from poised ones with H3K4me1 alone (Creyghton et al. 2010; Zentner et al. 2011), this indicates that the high-fidelity but half-methylated CpG sites may have resulted from cell type-specific methylation events. In addition, three- to fivefold enrichment at the binding sites of most TFs was also observed for the half-methylated CpG dyads with high fidelity (Supplemental Fig. S13B). Altogether, these results support that, for CpG dyads at similar methylation levels, those of higher methylation fidelity tend to be associated with functionally important regions. It further implied the vital importance for keeping methylation patterns, reliably maintained during cell cycles.

### Methylation level but not fidelity is correlated with the level of 5-hydroxymethylation

**Figure 6.**
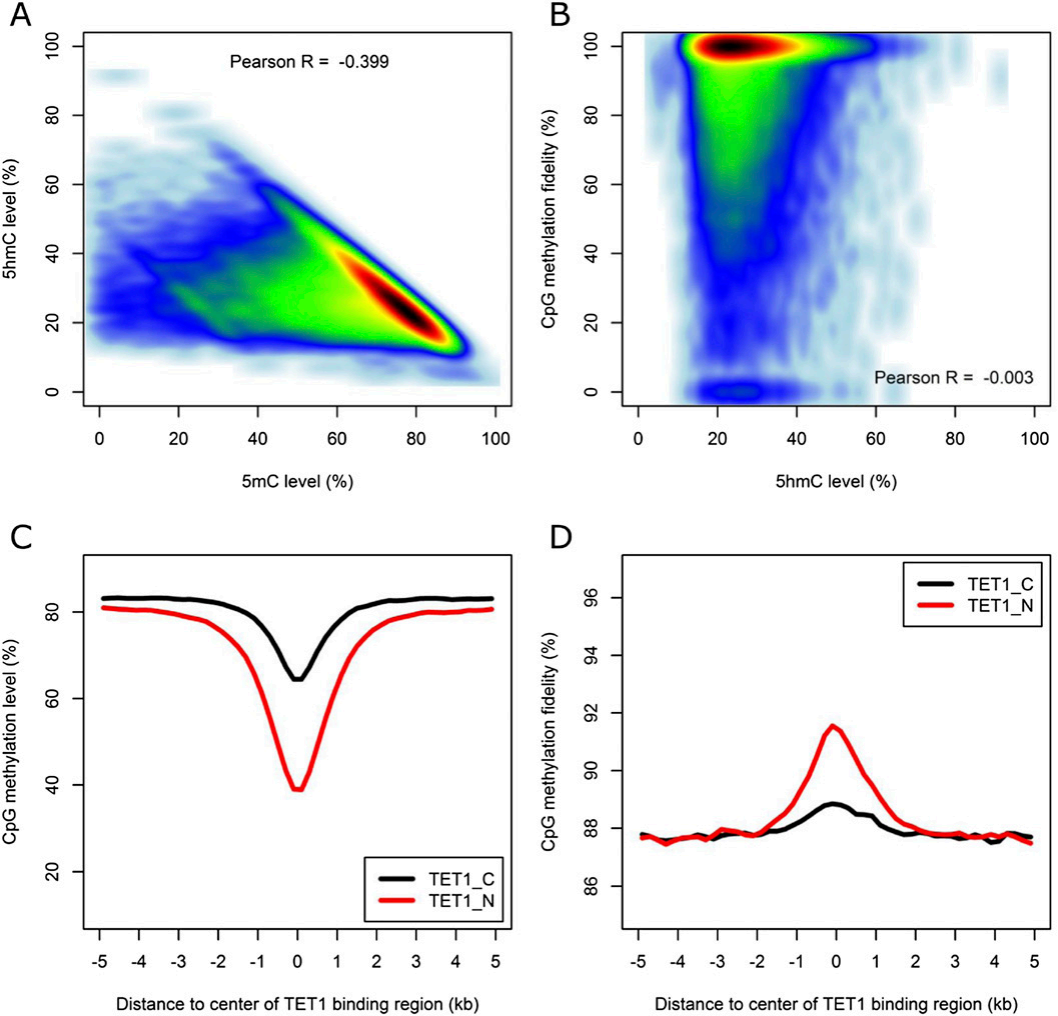
Relationship between DNA methylation and hydroxymethylation. (*A*) Scatter plot showing the relationship between 5-mC and 5-hmC. (*B*) Scatter plot showing the relationship between 5-hmC level and CpG methylation fidelity. (*C*,*D*) Profiles of methylation level and methylation fidelity surrounding TET1-binding regions calculated using 200-bp sliding windows.

Hydroxymethylation was proposed to be a mechanism for active demethylation to occur (Wu and Zhang 2010). To explore whether hydroxymethylation would increase the asymmetry of DNA methylation, we made use of the genome-wide hydroxymethylation data at single-base resolution determined for mouse ES-E14TG2a cells with TAB-seq in a recent study (Qin et al. 2012). We confirmed the correlation between 5-mC% determined in this study and 5-hmC% determined previously (Fig. 6A). Surprisingly, we found no correlation between the level of 5-hmC and methylation fidelity (Fig. 6B). We then classified CpG sites into three groups (with none, one, or two hmCs on both DNA stands). Compared with those with one or both cytosines hydroxymethylated, the CpG dyads with no hmC showed lower 5-mC + 5-hmC% (*P* < 2.2 × 10^−16^, Wilcoxon rank sum test) (Supplemental Fig. S14A), but no obvious difference in the average methylation fidelity was observed for these three groups (Supplemental Fig. S14B). In the present study, 5-hmC could not be distinguished from 5-mC with regular bisulfite treatment. Nevertheless, our result suggested that hydroxymethylated CpG are likely to be paired with either methylated or hydroxymethylated CpG on the complementary strand.

The hydroxymethylation reactions were catalyzed by TET enzymes. To investigate whether TET localization would contribute to the methylation fidelity, we integrated the TET1 ChIP-seq data (Williams et al. 2011) into the analysis. Decreased methylation levels and increased methylation fidelity were observed in TET-binding regions (Fig. 6C,D). This is consistent with the fact that many TET-binding regions tend to occur in promoter regions, which are frequently hypomethylated. As speculated previously (Williams et al. 2011), TET enzymes may assist in the maintenance of high methylation fidelity on these promoter regions through the removal of undesired methyl-groups introduced by stochastic methylation events. According to a previous study (Williams et al. 2011), ∼40% of hmC positive genes are bound by TET1 and other TET enzymes may also contribute to the generation of hmC in mouse ES cells. In addition, TET1 is enriched in the promoter regions while 5-hmCs are enriched in gene bodies. This may explain the difference in methylation observed for TET1-binding sites and 5-hmC enriched regions.

### Asymmetric non-CpG DNA methylation decreases during early differentiation

Non-CpG methylation has been shown to be abundantly present in embryonic stem cells but nearly depleted in somatic tissues (Lister et al. 2009; Ziller et al. 2011). The loss of non-CpG methylation occurs at the early stage of differentiation and during embryonic body formation (Ziller et al. 2011). We observed non-CpG methylation in various genomic regions with a mean methylation level of 0.4% and 0.2% for E14-d0 and E14-d6, respectively. Consistent with previous findings (Ziller et al. 2011), CpA methylation is the most frequent form of non-CpG methylation (Supplemental Fig. S15A,B). The low methylation level of non-CpG sites is consistent across different genomic regions in general, with the lowest level for CGIs, CGI shores, and promoters (Supplemental Fig. S15C).

Based on the single strand methylation data, mCHG was believed to be highly asymmetrical while 99% of mCG sites were methylated on both strands in the human cell lines (Lister et al. 2009). We examined the methylation pattern of two arms for each read pair and confirmed that CpG sites show highly correlated methylation patterns in two arms (Supplemental Fig. S16A,B). In contrast, the correlation of methylation statuses of non-CpG sites in the two arms is close to the baseline, that of the spike-in lambda DNA control (Supplemental Fig. S16C–F). Previous studies suggest possible dependence of CpA methylation on adjacent CpG methylation (Ziller et al. 2011). However, in our study, non-CpG methylation levels are only weakly correlated with the level of CpG methylation in surrounding regions (Supplemental Fig. S17). It remains an interesting question as to how the cytosines in non-CpG context are consistently methylated in a considerable fraction of the genome without templates for faithful propagation of the methylation states.

## Discussion

In this study, we present the first genome-scale analysis of hairpin bisulfite sequencing data for both differentiating and undifferentiated mES cells. During the early mES cell differentiation, we observed a global increase in both DNA methylation level and fidelity. In both E14-d0 and E14-d6 cells, DNA methylation varies across distinct genomic regions with promoter regions showing the lowest methylation levels and the highest methylation fidelities. In addition, we found that methylation fidelities follow a bimodal distribution. Given the high frequency of hemi-methylation of CpG dyads, particularly of those that are intermediately or highly (i.e., 50%–90%) methylated, we assumed that this phenomenon is of some important biological significance. For example, the small stretch (<100 bp) of hemi-methylated CpG dyads (particularly those located at distal regulatory elements such as enhancers) may serve as the origin of a switch from hypermethylation to hypomethylation, and vice versa, leading to the changes of chromatin statuses; as a result, expression of the surrounding genes would be dynamically regulated.

The methylation status for a given CpG site may be considered as the outcome of cross talk between the DNA molecule and the proteins/RNAs guiding the formation of the local chromatin structure and regulating the DNMTs’ activities. We investigated sequence features and gene-related attributes for four groups of CpG dyads: completely unmethylated, completely methylated, and half-methylated with 0% or 100% fidelity. In vitro, DNMT1 shows a high processivity on hemi-methylated DNA with low frequency of skipping sites (Vilkaitis et al. 2005; Goyal et al. 2006). It methylates hemi-methylated DNA with fidelity of >95% and no preference on flanking sequence. Similarly, we did not observe significant bias on flanking sequences of the two groups of half-methylated CpG dyads. However, the CpG dyads with high methylation fidelity tend to have higher levels of evolutionary conservation and are enriched in promoters and regions with high CpG density. Recently, Stadler and colleagues observed a class of low-methylated regions (LMRs) with methylation level ∼30% in mouse ES cells, which could be distal regulatory regions such as enhancers and TF-binding sites (Stadler et al. 2011). In this study, we found the majority of low-methylated CpGs tend to be with high methylation fidelity, and the CpG dinucleotides with high methylation fidelity are enriched at the TF-binding sites. Thus, the exploration of methylation fidelity may provide an additional indicator of the functional importance for partially methylated CpG sites.

The integration of various “omics” data revealed that both DNA methylation level and methylation fidelity are highly related to histone modifications and the binding of TFs. There are several potential “safeguard” mechanisms to ensure the high methylation fidelity in the promoter regions of active genes: (i) histone marks. Directed by RNA polymerase II, histone H3 and H4 acetylation and H3K4 methylation on CGIs prevent DNMT3L from accessing the chromatin and inhibit de novo DNA methylation (Guenther et al. 2007; Ooi et al. 2007; Cedar and Bergman 2009). On the other hand, H3K27 triple-methylation and the mono-ubiquitination on lysine 119 of histone H2A (uH2A) mediated by the Polycomb group (PcG) proteins, PRC2 and PRC1 Polycomb complexes, respectively, are positively correlated with the level of DNA methylation (Vire et al. 2006; Kallin et al. 2009). In this study, we observed that methylation fidelity is positively correlated with active histone modifications (H3K4me3, H3K4me2, and H3K27ac in particular) and negatively correlated with H3K27me3. (ii) TF binding. We examined the methylation profiles of genomic regions interacting with TFs and regulators. All the TF-binding regions demonstrate increased methylation fidelity to various degrees. For some TFs that maintain the undifferentiated ground state, such trends are diminished after cell differentiation. This may reflect the competition between the DNMTs and transcription machinery for promoter binding and DNMTs; DNMT3A/B in particular are continuously excluded from highly active promoters and the adjacent CGIs. (iii) Occupancy of TET enzymes. We observed an increase of methylation fidelity at the TET1-binding sites. This is consistent with previous reports that TET1 is particularly enriched on CpG-rich transcription start sites and potentially responsible for the removal of aberrant stochastic DNA methylation (Williams et al. 2011).

Lastly, we confirmed that the 5-hmC level and 5-mC level are anti-correlated as previously reported (Qin et al. 2012). To our surprise, the enrichment of 5-hmC does not result in the significant decrease in methylation fidelity, when no distinction was made between 5-hmC and 5-mC. This indicates the minimum pairing of unmethylated cytosine with 5-hmC or 5-mC at the 5-hmC-enriched sites. Therefore, the relatively low methylation fidelity in intermediately to highly (i.e., 50%–90%) methylated CpG dyads is not due to 5-hmC-mediated DNA demethylation. It also suggests that 5-hmC is a rather stable epigenetic mark as speculated in a recent study (Hahn et al. 2013). Instead of being removed actively within a cell cycle, it is more likely that 5-hmC is passively removed through replication, as shown in PGCs and pre-implantation embryos (Inoue and Zhang 2011; Branco et al. 2012; Hackett et al. 2013). Since DNMT1 prefers hemi-methylated (5-mC/C) substrates over hemi-hydroxymethylated ones (5-hmC/C) in vitro (Hashimoto et al. 2012), further study would be required to uncover how these sites maintain high levels of 5-hmC plus 5-mC during cell division. Recently, Fu and colleagues explored a hidden Markov model to capture substrate specificity and processivity of DNMTs with hairpin bisulfite sequencing data (Fu et al. 2012). We anticipate that the combination of such a statistical model, genome-wide hairpin bisulfite sequencing strategy described in this study and the experimental manipulation of DNMTs’ activities in the future, will provide an in-depth understanding of mechanisms implicated in the 5-mC and 5-hmC turnover.

## Methods

### Mouse ES cell culture and the induction of differentiation

Mouse ES cells (E14TG2a) were maintained on gelatin-coated dishes in ATCC-formulated Dulbecco’s Modified Eagle’s Medium (ATCC), supplemented with 0.1 mM of 2-mercaptoethanol, 10% fetal bovine serum (StemCell Technologies), 2 mM L-glutamine, 0.1 mM MEM nonessential amino acid, 100 U/mL penicillin, 10 µg/mlstreptomycin, and 10 ng/mL LIF (StemCell Technologies). The mES cells were passaged every 2 d at a ratio of 1:5 by washing with PBS, dissociating with 0.25% trypsin (GIBCO) for 3 min at 37°C, and resuspending in mES media. Media was changed daily. To induce differentiation, the mES cells were passaged and then cultured in ES cell culturing media without LIF. Media was changed every 2 d. The undifferentiated (E14-d0) and differentiating (E14-d6) states of mES cells were verified by SSEA-1 (stage-specific embryonic antigen-1) staining with StainAlive SSEA-1 Antibody (DyLight 488) (Stemgent) and quantitative RT-PCR analysis of relative expression levels of three major pluripotency factors including *Nanog*, *Sox2*, and *Pou5f1*.

### Hairpin bisulfite-seq library construction

Mouse ES cell genomic DNA was isolated using DNeasy Blood and Tissue kit (Qiagen). Ten-microgram mouse genomic DNA was spiked with 0.01% unmethylated cl857 Sam7 Lambda DNA (Promega) and sonicated to 200-bp fragments with Covaris S2 (AB). After purification (PureLink PCR Purification Kit, Invitrogen), DNA fragments were then subjected to end repair with the end repair enzyme mix (NEB), dA tailing using Klenow 3′–5′ exo- (NEB) with purification at each step. Ligation to biotin-modified hairpin adapter (P-CGCCGGCGGCAAG/iBiodT/GAAGCCGCCGGCGT) and Illumina TruSeq adapters were performed using T4 DNA ligase (NEB) overnight. DNA barcodes and “batch-stamp” may be introduced into hairpin adaptors to detect template redundancy and contaminant sequences (Miner et al. 2004). Adapter-ligated DNA was digested with MseI and MluCI (NEB) for 1 h at 37°C. After purification, DNA fragments were then pulled down using Dynabeads MyOne Streptavidin C1 beads (Invitrogen). Bisulfite conversion was performed using the EpiTect Bisulphite Kit (Qiagen). After bisulfite conversion, the single-stranded uracil-containing DNA was subjected to 12 cycles of PCR reaction with Illumina TruSeq PCR primers (with specific index) and 2.5 U Pfu TurboCx Hotstart DNA polymerase (Agilent) to recover enough DNA for sequencing. After purification, size selection of 400–600-bp fragments was conducted with LabChip XT DNA Assay (Caliper) to yield longer sequences that are more amenable for unambiguous mapping to the reference sequence. Libraries were sequenced using Illumina HiSeq 2000.

### Read processing and alignment

The paired-end reads are of 101 bp in length. For each read, adaptor and hairpin sequences were searched with cross_match. Additional searches on the 3′ end of sequence reads were conducted to eliminate any sub-string derived from hairpin sequence adaptor. After adaptor removal, sequence reads <40 bp were excluded from further analysis. The bisulfite conversion rate was estimated with spike-in control lambda DNA. To remove reads that are likely to be not bisulfite converted, read pairs with more than three methylated non-CpG in either arm were discarded as previously described (Lister et al. 2009).

Hairpin bisulfite sequencing enables the original genomic sequence to be recovered with the bisulfite converted sequences derived from two DNA strands. The following steps were taken to retrieve and map the original sequences onto the reference genome: (i) the two arms for each read pair were first subjected to C→T and G→A conversion, and then globally aligned using the Needleman-Wunsch algorithm; (ii) the sequence reads with identity <90% between two arms were discarded; (iii) after trimming the overhangs of the aligned sequences, original sequences were recovered according to the alignment information; (iv) the recovered original sequences were mapped to mouse genome (GRCm38/mm10, random sequences and unassembled chromosomes were excluded) using Bowtie (Langmead et al. 2009) with parameters (-n 2 –l 40 –k 1 –m 1 --best), and only uniquely mapped reads were retained.

The methylation patterns for all cytosines were extracted based on the mapping result and the raw sequences. To eliminate the influence of SNP on the data analysis, the methylation pattern calling for nucleotide bases inconsistent with reference genome was masked. Finally, a common data set between E14-d0 and E14-d6 for cytosines with ≥10× sequencing depth was obtained. Statistics including the total uniquely mapped read number, genome coverage, cytosine coverage, CpG coverage, and the average sequencing depth were summarized in Supplemental Table S1.

### Genome annotation

Based on NCBI assembly GRCm38/mm10, the annotations for genomic regions, including transcripts, repetitive elements, and CpG island, were downloaded from UCSC Genome Browser (Fujita et al. 2011). Promoters were arbitrarily defined as regions 1 kb upstream of each transcript. 5′ UTR, exon, intron, and 3′ UTR were defined according to previous studies (Lister et al. 2009). Intergenic regions were defined as regions not falling into the 10-kb flanking genic regions (Molaro et al. 2011). Several major types of repetitive elements, including SINE, Simple_repeat, LINE, LTR, Low_complexity, DNA, and Satellite, were analyzed in this study. When analyzing methylation patterns along gene-associated regions, each element was divided into 20 equally sized bins, and the pattern for each bin was calculated and averaged for plotting.

### The determination of methylation level and fidelity

Methylation level (ML) for each C site shows the fraction of methylated Cs, and is defined as
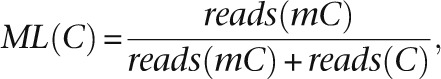
where *reads(mC)* is the number of reads with methylated Cs and *reads(C)* is the number of reads with unmethylated Cs. The counts for the same CpG dyad were merged. Calculated ML was further corrected with the bisulfite nonconversion rate according to previous studies (Lister et al. 2013). Given the bisulfite non-conversion rate *r*, the corrected ML was estimated as
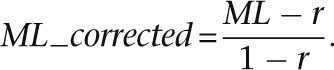


The minimum corrected ML was set as zero. Cytosines in different genome contexts were corrected separately. The nonconversion rates for CpG, CHG, and CHH were estimated as 1.03%, 1.18%, and 1.12% for E14-d0, and 1.06%, 1.13%, and 1.23% for E14-d6, respectively.

CpG dyads can be classified as unmethylated, asymmetrically methylated, or symmetrically methylated, based on the methylation pattern of the two Cs on different strands. The methylation fidelity (MF) for a CpG dyad is defined as
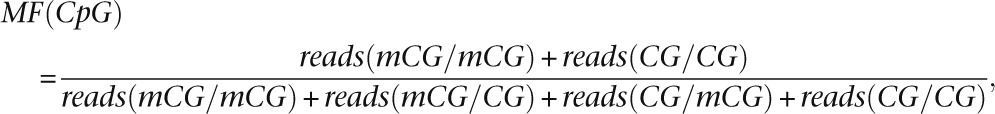
where reads(mCG/mCG) is the number of fully methylated CpG dyads detected, reads(CG/CG) is the number of fully unmethylated CpG dyads detected, and *reads(mCG/CG)* and *reads(CG/mCG)* is the number of hemi-methylated CpG dyads detected for a given CpG site.

When calculating the methylation level for a given genomic region, we first determined the number of mCG/mCG, mCG/CG, CG/mCG, and CG/CG, then used the function aforementioned to calculate a weighted methylation level (Schultz et al. 2012). Similar calculations were performed for the methylation fidelity for a given genomic region. Significantly methylated Cs were identified by using binomial distribution as previously reported (Lister et al. 2009). The probability *p* in the binomial disbribution B(*n*,*p*) was estimated from the unmethylated Lambda genome (it equals the nonconversion plus sequencing error rate). Cs in CpG, CHG, and CHH context were analyzed separately, and 0.01 was used as the FDR cutoff to determine significantly methylated Cs. Base frequencies surrounding methylated non-CpG sites were illustrated using WebLogo (Crooks et al. 2004).

### Analysis of RNA-seq data

Total RNA was extracted with the miRNeasy extraction kit (Qiagen). RNA-seq libraries were constructed according to Illumima protocol and sequenced with the Illumina Hiseq 2000. Using TopHat (version 2.0.3), all the 101-bp paired-end reads were mapped to the mouse reference genome (GRCm38/mm10) (Trapnell et al. 2009). Genome annotation files with GTF format for Known Genes were downloaded from UCSC. Reads per kilobase of transcript per million reads (RPKM) values were calculated for each gene using the Cufflinks software (version 2.0.2) with default parameters (Trapnell et al. 2010) and normalized using the quantile method. Genes were classified into five groups according to their expression levels. Specifically, genes with no detectable expression were classified as group 1, and the remaining genes were classified as four equally sized groups. Methylation level and fidelity surrounding the TSSs of different groups of genes were calculated in bin windows of 200 bp.

### Analysis of ChIP-seq data for histone modification, histone variant, transcription factors, and TET

ChIP-seq data for several types of histone modifications (H3K27ac, H3K27me3, H3K4me1, H3K4me2, H3K4me3, and H3K36me3), histone variant (H2A.Z), and TFs or regulators (NANOG, POU5F1, SOX2, ESSRB, ZFX, KLF4, MYC, MYCN, CTCF, SUZ12, TFCP2L1, STAT3, and E2F1) published by previous studies (Mikkelsen et al. 2007; Chen et al. 2008; Xiao et al. 2012) were downloaded from NCBI Gene Expression Omnibus (GEO). For TFs and regulators, the downloaded ChIP-seq peak-calling results were used directly. The ChIP-enriched peaks were identified by SICER (Zang et al. 2009) for histone modifications and variant (window size = 200, FDR = 0.001, gap size = 600 for H3K36me3, and gap size = 200 for others), and by MACS (Zhang et al. 2008) for TET1 with parameters: -g mm -*P*-value 1 × 10^−5^.

### Analysis of TAB-seq data for 5-hydroxymethylation

The single-base resolution 5-hmC data were generated with TET-assisted bisulfite sequencing (TAB-seq) strategy in a previous study (Qin et al. 2012). The 5-hmC levels were adopted and the coordinates of called 5-hmCs were converted to a GRCm38/mm10 version by using the UCSC liftOver tool. A set of CpG sites with both hairpin bisulfite sequencing data and called 5-hmCs was identified for analysis.

## Data access

The data generated in this study have been submitted to the NCBI Gene Expression Omnibus (GEO; http://www.ncbi.nlm.nih.gov/geo/) under accession number GSE48229. A summary of other data sources including TAB-seq and ChIP-seq for mouse embryonic stem cell line E14TG2a has been provided in Supplemental Table S2.
